# Unilateral thalamic infarction presenting as vertical gaze palsy: a case report

**DOI:** 10.1186/1752-1947-5-535

**Published:** 2011-10-31

**Authors:** Muhib Khan, Christos Sidiropoulos, Panayiotis Mitsias

**Affiliations:** 1 Department of Neurology, Henry Ford Hospital, 2799 West Grand Boulevard, Detroit, MI, USA

## Abstract

**Introduction:**

Vertical gaze palsy is a recognized manifestation of midbrain lesions. It rarely is a consequence of unilateral thalamic infarction.

**Case presentation:**

We report the case of a 48-year-old African-American woman who presented to our facility with vertical gaze palsy and evidence of left medial thalamic infarct on diffusion-weighted imaging without coexisting midbrain ischemia. The etiology of infarct was determined to be small vessel disease after extensive investigation.

**Conclusions:**

This report suggests a possible role of the thalamus as a vertical gaze control center. Clinicoradiological studies are needed to further define the role of the thalamus in vertical gaze control.

## Introduction

Vertical gaze palsy is usually associated with lesions of the mesencephalic rostral interstitial nucleus of the medial longitudinal fasiculus, the interstitial nucleus of Cajal, the posterior commissure and the peri-aqueductal gray matter. Rarely, vertical gaze palsies can be a manifestation of paramedian thalamic infarction [1-3]. Here, we describe the case of a patient presenting with upward gaze palsy secondary to isolated medial thalamic infarct.

## Case presentation

**Figure 1:**
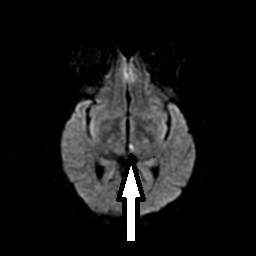
**Diffusion-weighted image showing an acute ischemic infarct in the left medial thalamus**.

**Figure 2:**
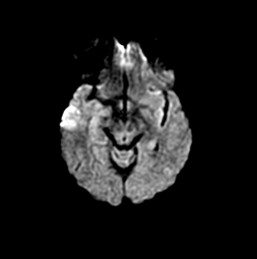
**T2-weighted image of the left medial thalamic infarct**.

**Figure 3:**
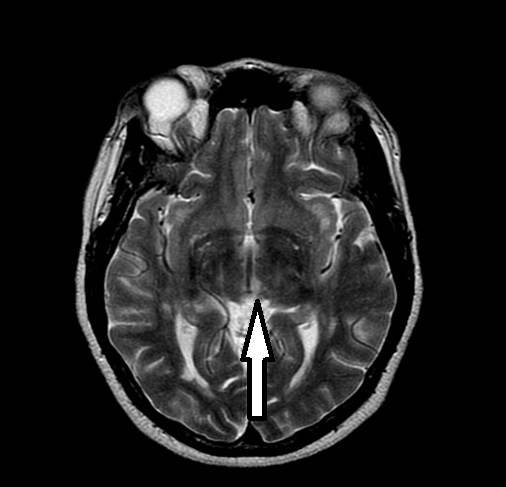
**T2 fluid attenuated inversion recovery (FLAIR) image of the left medial thalamic infarct**.

**Figure 4:**
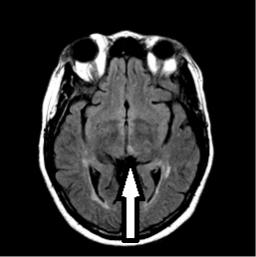
**Diffusion-weighted image of midbrain with no ischemia**.

A 48-year-old African-American woman with diabetes, hypertension and hyperlipidemia presented to our facility with acute onset of dizziness and vertical diplopia. A physical examination revealed upward gaze paresis, which could be overcome by the doll's eye maneuver and skew deviation of the right eye. A magnetic resonance imaging (MRI) scan, which was performed 12 hours after the onset of symptoms, showed an acute left paramedian thalamic infarct (Figure 1, Figure 2 and 3) without associated midbrain lesions (Figure 4), and a chronic right cerebellar infarct. Stenosis of the right vertebral artery at the C4 transverse foramen secondary to extrinsic osteophyte compression was seen on magnetic resonance angiography and confirmed by catheter angiography. There was slight worsening of the degree of narrowing when the head was rotated to the right, but there was no flow limitation during the catheter angiography. No dissection of the vertebral arteries was noticed.

A transesophageal echocardiogram revealed an ejection fraction of 55% with no atrial or ventricular thrombus or intracardiac shunt. The etiology of stroke was thought to be due to small vessel disease secondary to uncontrolled diabetes and hypertension. Treatment with aspirin, simvastatin, and tight hypertension and diabetes control was initiated. No neuropsychological testing was performed.

## Discussion

This is a report of a rare acute left medial thalamic infarction manifesting as supranuclear upward gaze palsy and skew deviation. A few previous reports have described vertical gaze palsies in patients with unilateral or bilateral paramedian thalamic infarction, but attributed the gaze palsy to a coexisting midbrain lesion [4], identified primarily at autopsy. An important clinical feature in our patient was the skew deviation, which has been reported with thalamic infarctions [5].

The medial thalamus is supplied by perforating branches arising from the basilar communicating artery and posterior cerebral arteries. The midbrain is spared because the superior and inferior paramedian mesencephalic arteries arise separately from each other from the basilar communicating artery [6].

The supranuclear pathways involved in vertical gaze are not well understood. Studies on primates reveal that the frontal eye fields traverse the medial thalamus [7]. Also, the internal medullary lamina has reciprocal connections with the frontal and supplementary eye fields. Interruption of supranuclear fibers as they traverse the medial thalamus en route to the pretectal and prerubral areas [3,8] could possibly lead to vertical gaze paresis. The mechanism of vertical gaze paresis with unilateral lesions is uncertain but we can speculate on the possibility of decussation of the frontobulbar fibers in the medial thalamus, as suggested in a case series of thalamic infarctions presenting as vertical gaze palsies [9]. The neuroimaging study results from our patient revealed no midbrain lesion. There has been a previous case reported of transient vertical gaze palsy with resolution of symptoms within three hours, highlighting the role of the thalamus in vertical gaze [10].

## Conclusions

The combination of vertical gaze paresis and skew deviation, previously believed to be pointing to a brainstem lesion, may now be attributed to a broader spectrum of anatomical areas. However, more cases correlating MRI findings with clinical presentations as attempted by Weidauer *et al*. need to be studied in order to establish the role of the thalamus in vertical gaze as either a crossroads or an actual control center [11].

## Consent

Written informed consent was obtained from the patient for publication of this case report and any accompanying images. A copy of the written consent is available for review by the Editor-in-Chief of this journal.

## Competing interests

The authors declare that they have no competing interests.

## Authors' contributions

MK was involved in the diagnosis and treatment of our patient, and wrote the manuscript. CS was involved in the diagnosis of our patient and helped with revising the manuscript. PS was involved in the diagnosis and management of our patient and helped in revising the manuscript. All authors read and approved the final manuscript.
